# Prevalence of metabolic syndrome among adults treated at a district hospital outpatient department

**DOI:** 10.4102/safp.v66i1.5959

**Published:** 2024-09-30

**Authors:** Cara van Jaarsveldt, Tlholohelo Jabari, Elrine Zwarts, Simone Färber, Yothando Sikuza, Heinrich Schilling, Sebastiaan Pauw, Elizabeth Klein, Cornel van Rooyen, Gina Joubert, Chantelle C. van der Bijl

**Affiliations:** 1 Department of Family Medicine, Faculty of Health Sciences, University of the Free State, Bloemfontein, South Africa; 2 Department of Biostatistics, Faculty of Health Sciences, University of the Free State, Bloemfontein, South Africa

**Keywords:** metabolic syndrome, non-communicable diseases, hypertension, diabetes, obesity

## Abstract

**Background:**

Metabolic syndrome (MetS) is a collection of risk factors, including hypertension, high fasting blood glucose, high fasting triglyceride and low high-density lipoprotein (HDL) cholesterol levels that may increase the risk for cardiovascular disease and type 2 diabetes. The study aimed to determine the prevalence of MetS among adults attending a Free State district hospital’s outpatient department.

**Methods:**

A cross-sectional study included a consecutive sample of consenting patients 18 years and older from 18 October 2021 to 19 November 2021. Patients’ waist circumference was measured, and data were extracted from patients’ files.

**Results:**

The 409 participants were predominantly females (64.2%). The median age was 60 years. Triglyceride and HDL cholesterol levels were available for 27.4% and 26.9% of patients, respectively. Of the 278 (68.0%) patients with sufficient information to determine their MetS status, 187 (67.3%) had MetS. Of the males with sufficient information, 49.1% (*n* = 56/114) had MetS compared to 79.9% (*n* = 131/164) of the females with sufficient information (*p* < 0.001). The age group 60–79 years had the highest prevalence (76.7%, *p* < 0.001). In all race groups, at least two-thirds of patients had MetS (*p* = 0.831).

**Conclusion:**

Incomplete patient notes and failure to do investigations led to a third of patients not having sufficient information to determine their MetS status. In patients with sufficient information, a high prevalence of MetS was found.

**Contribution:**

This study highlights the challenges of determining MetS retrospectively in an outpatient population and the need for completeness of medical note keeping and routine investigations in high-risk patients. It also notes the high prevalence of MetS.

## Introduction

Metabolic syndrome (MetS) is the umbrella term for the collection of conditions that may increase the risk of developing cardiovascular disease, stroke and type 2 diabetes.^1^ The World Health Organization (WHO) defines MetS as a combination of conditions such as abdominal obesity, insulin resistance, hypertension and hyperlipidaemia.^2^ According to the International Diabetes Federation (IDF), a person is diagnosed with MetS if (1) abdominally obese, with a waist circumference of ≥ 94 cm for men and ≥ 80 cm for women, (2) presenting with two or more of the other risk factors: high fasting blood glucose (FBG), elevated blood pressure (BP), low high-density lipoprotein (HDL) cholesterol and/or raised triglyceride levels.^3^ A person, therefore, has MetS if they present with at least three of the five risk factors, including abdominal obesity. Refer to Table 1 for a concise summary between normal levels and risk factor levels.^3,4,5,6,7,8,9^

**TABLE 1:** Normal and risk factor measurements of metabolic syndrome components.

Component	Normal levels†	Risk factor†
**Waist circumference (cm)**		
Women	Dependent on population and country-specific definitions	≥ 80
Men		≥ 94
Fasting TG level (mmol/L)	< 1.7	≥ 1.7
**HDL cholesterol level (mmol/L)**		
Women	> 1.5	< 1.3
Men	> 1.3	< 1.0
Blood pressure (mmHg)	115–120/25–80	≥ 130/85
FBG level (mmol/L)	< 5.6	≥ 5.6

*Source*: International Diabetes Foundation. The IDF consensus worldwide definition of the metabolic syndrome [document on the internet]. Belgium: IDF; 2006 [cited 2022 Feb 25]. Available from: https://idf.org/media/uploads/2023/05/attachments-30.pdf

FBG, fasting blood glucose; HDL, high-density lipoprotein; TG, triglyceride.

† , Compiled from references.^3,4,5,6,7,8,9^

Metabolic syndrome has become a public health concern worldwide.^10^ Data from Statistics South Africa in 2018 showed that diabetes (5.9%), hypertensive disease (4.5%), ischaemic heart disease (3.0%) and other forms of heart disease (5.1%) were ranked among the 10 leading natural causes of death.^11^ Persons meeting the criteria for MetS are more likely to develop these diseases, which would contribute to a higher mortality level, therefore making it an important public health issue that needs to be addressed.^1^ Other clinical conditions associated with MetS include cancer, polycystic ovarian syndrome, neurological disorders and non-alcoholic steatohepatitis.^12^ Pathology mainly targets the pancreas, liver and cardiovascular system.^13^

The human immunodeficiency virus (HIV) and its effects on MetS is a subject of research not often pursued. In patients with HIV, an increase in triglyceride levels and a decrease in HDL cholesterol levels were found in patients on antiretroviral therapy (ARVT).^14,15^ A systematic review conducted in sub-Saharan Africa in 2024 found that a combination of common symptoms of HIV and side effects of ARVT could potentially aggravate MetS.^16^ There have been a few contradictions in research about the relationship between HIV and the size of an individual’s waist circumference.^17,18^

Leading a sedentary lifestyle often aggravates the cluster of risk factors of MetS.^19^ The WHO estimated in 2016 that 23% of men and 32% of women worldwide were not reaching the recommended 150 min of physical activity a week.^19^ The numbers in South Africa were bleaker, where 29% of men and 47% of women reported low physical activity.^19^ Being sedentary often leads to individuals becoming overweight, and obesity may be causally related to developing diabetes and hypertension.^20^ A study conducted by the University of the Free State found that an increase in the prevalence of MetS was because of physical inactivity and poor control of pre-existing lifestyle-related risk factors such as hypertension (e.g. not taking medication).^4^

It is estimated that 20% – 25% of the global population has MetS, with ethnic and gender differences in the prevalence.^21^ Numerous studies have found that women are more likely to develop MetS than men.^21,22,23,24^ Most MetS research has been conducted among European populations, even though they comprise less than 20% of the world’s population.^22,23^ Studies found that socially and economically deprived people are more likely to develop MetS.^25^ Reasons for this may include a lack of adequate healthcare and health education among people of a lower socio-economic status.^25^ In South Africa, a low- and middle-income country (LMIC), a study published in 2017 found that the prevalence of MetS in the Eastern Cape for adults attending clinics was 21.8%, with a prevalence of 15.6% in males and 24.8% in females.^26^

Racial demographics play a significant role in abdominal waist circumference and different optimal waist circumference cut-offs exist for different ethnic groups.^21^ There are limitations regarding the evidence of the cause of obesity and MetS in the African population.^27^ The African population has a resource-constrained environment, with an inadequate determination of waist circumference for people living on the continent.^28^ By doing more research in the field and ensuring that clinicians accurately measure waist circumference, misdiagnosis of patients can be prevented.^23^

Metabolic syndrome is often not acknowledged as a serious condition, especially in South Africa.^26,28^ There had been little research performed in South Africa, especially in comparison to other countries. It was decided to explore the prevalence of MetS in adults after identifying this gap in the literature. Hypertension and type 2 diabetes remain common non-communicable diseases and are a significant concern in our community, as both conditions increase the risk of developing cardiovascular disease.^3,29^ It is important to identify these risk factors that are often already present in outpatients so that they can be described in a South African context and thus prevent further sequelae of MetS.

### Aim and objectives

This research aimed to determine the prevalence of MetS among adults who presented at the Outpatient Department (OPD) of the National District Hospital, Free State. In order to place this finding in context, the objectives were to describe the sociodemographic profile and clinical conditions (including tuberculosis, HIV, hypertension and type 2 diabetes) of the study participants. Associations between sociodemographic characteristics and clinical conditions were also investigated.

## Research methods and design

### Study design and setting

This was a cross-sectional study with descriptive and analytic components. The study was conducted at the National District Hospital, one of the three district hospitals in the Mangaung Metropolitan Municipality. The hospital has a bed capacity of 154 and serves patients from the surrounding areas. The OPD specifically serves patients receiving chronic care. Three to four doctors consult the patients.

### Study population and sampling strategy

The study population included all patients, 18 years and older, who presented at the OPD for treatment from 18 October 2021 to 19 November 2021. On average, 40 patients were seen at the OPD per day during this study. Consecutive sampling included the entire study population, fulfilling inclusion criteria during this period. Exclusion criteria were patients in wheelchairs, missing patient files and patients transferred to the casualty department because of ill health.

### Data collection

The measuring tool was a data collection sheet developed by the researchers and included three sections. The first section dealt with the study participants’ sociodemographic features, for example, age, sex, race and employment. The second section dealt with the criteria and measurements for MetS, such as abdominal circumference, TG levels, HDL levels, BP, FBG levels, height and weight. The height and weight measurements were used to calculate body mass index (BMI). Section three assessed the participants’ clinical conditions, for example, HT, DM, HIV and TB.

At the start of each day, the researchers called on patients in the waiting room and briefly explained what the study entailed. Patients who showed interest in participating in the study were taken to consultation rooms where they read the consent form, or it was read to them. The consent forms were provided in Afrikaans, English or Sesotho. Willing patients signed the consent form, and it was co-signed by a witness. As the routine assessment sheet at the OPD did not include waist circumference measurement, the researchers then measured the participants’ waist circumference. It was measured while the patient was seated at a midpoint between the ribcage and the iliac crest of the pelvic bone. As such, standardisation was ensured between the researchers. The researchers supplied the tape measures. The patient then returned to the waiting room for their consultation with the doctor.

After all the patients were consulted on a given day, the research team members captured the relevant information from the patients’ files. The researchers numbered the green sticker on the file to prevent duplication, that is, to prevent the file from being audited twice. The number on the sticker was also noted on the data collection sheet. If the patient did not have recorded lipogram results in the file, the researchers searched on the NHLS-Labtrak (National Health Laboratory Service) for previous results using the patient’s full name, date of birth or hospital number. Results obtained had no time cut-off restriction from date of data collection. No information on the patient’s current medications was taken, including lipid-lowering medication. Data collection took place from 19 October 2021 to 19 November 2021.

Ten participants were included in the pilot study on 18 October 2021 to review the effectiveness of the data sheet and the practicality of the study. As no changes were made to the data collection method, data collection for the main study started the following day and the data of these 10 participants were included in the main study.

### Data analysis

The researchers transferred the information on the data collection sheets to a Microsoft Excel spreadsheet. The data were analysed by Department of Biostatistics, Faculty of Health Sciences of the University of the Free State using SAS software, version 9.4. Numerical variables were summarised by median and interquartile range (IQR). Categorical data were reported as frequencies and percentages. Associations between categorical variables were assessed using Chi-square or Fisher’s exact tests.

To determine the prevalence of MetS in the study population, the patients had to meet at least three (waist circumference must be one of the three criteria) of the five IDF criteria^3^:

- Abdominal obesity (≥ 94 cm in men; ≥ 80 cm for women).
- Raised fasting triglycerides (≥ 1.7 mmol/L) and/or reduced HDL cholesterol (< 1.0 mmol/L in men; < 1.3 mmol/L in women) and/or elevated BP (systolic BP ≥ 130/85 mmHg or individuals already on treatment for hypertension) and/or raised FBG (≥ 5.6 mmol/L or individuals previously diagnosed with type 2 diabetes).

### Ethical considerations

Ethical clearance to conduct this study was obtained from the University of the Free State Health Sciences Research Ethics Committee (No. UFS-HSD2021/0442/2809-0001). Permission to perform the study at the National District Hospital was obtained from the Free State Department of Health.

Consent forms were read by or to the participants in their preferred language (Afrikaans, English or Sotho) and signed by participants to indicate informed voluntary consent. The trace form linking the patient file number and their study number was kept confidential to protect personal information. The electronic file was password coded, and only the researchers, supervisors and the statistician had access to the data. All data were handled confidentially.

## Results

A total of 769 patients was seen by the OPD doctors from 18 October 2021 to 19 November 2021. Only 409 (53.2%) patients gave informed consent to partake in the study.

### Sociodemographic features

Two-thirds (64.2%) of the study population were females (Table 2). The median age of the patients was 60 years (IQR: 50–68 years). Almost half (47.1%) were in the 60–79 years age range. The majority of the patients were black people (69.0%) and 44.5% were unemployed.

**TABLE 2:** Sociodemographic profile of patients (*n* = 409).

Variable	*n*	%
**Gender (*n* = 408)**		
Male	146	35.8
Female	262	64.2
**Age group (years) (*n* = 408)**		
18–39	48	11.8
40–59	155	38.0
60–79	192	47.1
≥ 80	13	3.2
**Race**		
Black people	282	69.0
White people	99	24.2
Mixed race people	25	6.1
Indian people	3	0.7
**Employment status**		
Employed	65	15.9
Unemployed	182	44.5
Pensioner	161	39.4
Other	1	0.2

### Clinical conditions and components of metabolic syndrome

Table 3 shows components of MetS, other conditions and BMI distribution.

**TABLE 3:** Components of metabolic syndrome and other conditions.

Variable	*n*	%
Abdominal obesity (*n* = 408)	337	82.6
Raised triglycerides (*n* = 112)†	38	33.9
Reduced HDL cholesterol (*n* = 110)†	40	36.4
Raised BP or diagnosed with hypertension (*n* = 409)	347	84.8
Raised FBG or diagnosed with diabetes (*n* = 339)†	194	57.2
Tuberculosis (*n* = 331)	11	3.3
HIV (*n* = 86)	32	37.2
**BMI (kg/m^2^) (*n* = 397)**		
Underweight < 18	19	4.8
Normal weight 19–24.9	81	20.4
Overweight 25–29.9	107	27.0
Obese – Class I 30–34.9	98	24.7
Obese – Class II 35–39.9	48	12.1
Obese – Class III ≥ 40	44	11.1

HIV, human immunodeficiency virus; BMI, body mass index; BP, blood pressure; FBG, fasting blood glucose; HDL, high-density lipoprotein; TG, triglyceride.

† , Of the 337 patients with abdominal obesity, 72.1% had missing values for triglycerides, 72.7% for HDL cholesterol and 15.7% for FBG or diabetes.

The hypertension status of all patients could be determined, with the majority (84.8%) classified as hypertensive. Information regarding diabetes diagnosis or full blood count was available for 82.9% of patients. The final number of patients who had an elevated FBG level (≥ 5.6 mmol/L) or were previously diagnosed with diabetes was 194 (57.2%). Of these, 43 (22.2%) patients were not diagnosed with diabetes but satisfied the inclusion criteria with elevated FBG levels. It was observed in 151 patient files that they were already diagnosed with diabetes. For 16 of these 151 patients, the FBG levels were not measured or recorded, 120 patients had values in the normal range and 15 patients had elevated levels. Of the patients with unknown or undiagnosed diabetes status, 70 did not have FBG values.

Values for HDL cholesterol and triglyceride were only available for 26.9% and 27.4% of patients, respectively, with approximately a third of these patients qualifying as having the specific component of MetS.

Only 20.4% of the participants had normal BMI values. More than a quarter (27.0%) of the patients fell in the overweight category and almost half (47.9%) had a BMI classified as obese (class I, II and III).

The HIV status of 79.0% of the patients was not indicated in their files. Of those who did have an HIV status indicated, 37.2% were HIV positive. Of the patients for whom the data were available, 3.3% were previously diagnosed with tuberculosis.

Table 4 shows the prevalence of hypertension, elevated FBG or diabetes and abdominal obesity among the different genders, age and race groups. Hypertension was significantly associated with age group (*p* < 0.001) with more than 95% of patients 60 years and older having hypertension. No significant differences regarding hypertension were found between genders (*p* = 0.666) or race groups (*p* = 0.740). Diabetes was significantly associated with race group (*p* < 0.001) and age group (*p* < 0.001) with black patients and patients in the age group 60–79 years having the highest prevalence of diabetes. No significant differences were found between genders regarding diabetes (*p* = 0.823). Significantly more females than males had abdominal obesity (*p* < 0.001). Abdominal obesity was significantly associated with age group (*p* = 0.002) with patients in the age group 18–39 years least likely to have abdominal obesity. No significant differences regarding abdominal obesity were found between race groups (*p* = 0.554).

**TABLE 4:** Prevalence of hypertension, diabetes and abdominal obesity per gender, age group and race.

Variable	*n*	%	*p*
**Hypertension**			
**Gender**			
Male (*n* = 146)	122	83.6	0.666
Female (*n* = 262)	224	85.5	-
**Age group (years)**			
18–39 (*n* = 48)	24	50.0	< 0.001
40–59 (*n* = 155)	122	78.7	-
60–79 (*n* = 193)	188	97.4	-
≥ 80 (*n* = 13)	13	100.0	-
**Race**			
Black people (*n* = 282)	242	85.8	0.740
White people (*n* = 99)	81	81.8	-
Mixed race people (*n* = 25)	21	84.0	-
Indian people (*n* = 3)	3	100.0	-
**Elevated FBG or diagnosed with diabetes**			
**Gender**			
Male (*n* = 122)	71	58.2	0.823
Female (*n* = 216)	123	56.9	-
**Age group (years)**			
18–39 (*n* = 35)	15	42.9	< 0.001
40–59 (*n* = 133)	71	53.4	-
60–79 (*n* = 160)	106	66.3	-
≥ 80 (*n* = 11)	2	18.2	-
**Race**			
Black people (*n* = 236)	146	61.9	< 0.001
White people (*n* = 77)	32	41.6	-
Mixed race people (*n* =23)	13	56.5	-
Indian people (*n* = 3)	3	100.0	-
**Abdominal obesity**			
**Gender**			
Male (*n* = 146)	95	58.2	< 0.001
Female (*n* = 262)	242	56.9	-
**Age group (years)**			
18–39 (*n* = 48)	30	62.5	0.002
40–59 (*n* = 155)	128	82.6	-
60–79 (*n* = 192)	167	87.0	-
≥ 80 (*n* = 13)	12	92.3	-
**Race**			
Black people (*n* = 282)	229	81.2	0.554
White people (*n* = 98)	85	86.7	-
Mixed race people (*n* =25)	20	80.0	-
Indian people (*n* = 3)	3	100.0	-

FBG, fasting blood glucose.

### Prevalence of metabolic syndrome

Overall, 278 (68.0%) patients had sufficient information to determine their MetS status. Most of these patients (*n* = 187, 67.3%) had MetS. Table 5 depicts the prevalence of MetS in the different gender, age and race groups. Of the 114 males with sufficient information, 56 (49.1%) satisfied the criteria for MetS compared to 131 (79.9%) of the 164 females with sufficient information (*p* < 0.001). Metabolic syndrome was significantly associated with age group (*p* < 0.001) with the age group 60–79 years having the highest prevalence (76.7%). In all race groups, at least two-thirds of patients had MetS with no significant difference between the race groups (*p* = 0.831).

**TABLE 5:** Prevalence of metabolic syndrome per gender, age group and race.

Variable	*n*	%	*p*
**Gender**	-	-	< 0.001
Male (*n* = 114)	56	49.1	-
Female (*n* = 164)	131	79.9	-
**Age group (years)**	-	-	< 0.001
18–39 (*n* = 29)	10	34.5	-
40–59 (*n* = 95)	60	63.2	-
60–79 (*n* = 146)	112	76.7	-
≥ 80 (*n* = 8)	5	62.5	-
**Race**	-	-	0.831
Black people (*n* = 199)	133	66.8	-
White people (*n* = 59)	39	66.1	-
Mixed race people (*n* = 17)	12	70.6	-
Indian people (*n* = 3)	3	100.0	-

## Discussion

This research aimed to determine the prevalence of MetS among adults who presented at the OPD of the National District Hospital, Free State. Metabolic syndrome has grown in prevalence in South Africa and worldwide, especially in developing countries.^26,28^ The IDF criteria were used for this study, where high waist circumference and any other two criteria had to be present.^3^ Other organisations, such as the National Cholesterol Education Programme/Adult Treatment Panel III (NCEP/ATP III) and WHO, define the presence of any three or more of the criteria as MetS.^1,30^ The results of this study show that 187 of 278 participants with sufficient information satisfied the IDF criteria for MetS, which is 67.3%. This percentage is much higher than the previously estimated prevalence in South Africa of 21.8% for the general population.^28^ However, this may be because many of the patients presenting at OPD are older (i.e. more likely to present with the risk factors for MetS) and being treated for non-communicable diseases such as diabetes and hypertension.

The prevalence of MetS in women was significantly higher than in males, as has been found in previous studies.^22,23,24,28^ A study conducted in the United States determined that between 2007 and 2012, the prevalence of MetS was 27% and 35% for African American men and women, respectively.^31^ The prevalence of the white American population was 35% for men and 36% for women.^31^ No significant differences in MetS prevalence were found in our study regarding race groups.

The age group 60–79 years had the highest prevalence of MetS. A study examining adults presenting at healthcare facilities in the Eastern Cape, South Africa, saw a prevalence of 49.3% in participants 56 years and older.^26^ Another South African study focusing on the farmworker community in the Boland district found the highest prevalence of MetS in the 40–49 years age range.^32^ Reasons for this may include a lack of physical activity with old age and a worsening of the risk factors for MetS because of natural ageing processes.

The participants were predominantly female (64.5%) compared to the 51.1% females in the South African population of 2020.^33^ Most of the study participants were black people (69%), followed by white people, mixed race people and Indian people, consistent with Statistic South Africa data.^34^ Most participants fell within the 60- to 70-year age group, and 44.5% were unemployed (possibly because most participants were retired). The study sample thus consisted of predominantly female and elderly participants, who are more likely to present with MetS according to the findings of multiple studies.^22,23,24,28,35,36^ This stresses the need to investigate risk factors and guide healthcare interventions to address MetS in this setting.^37^

While not described in the IDF’s definition of MetS criteria, the BMI of each participant was determined and categorised.^3^ The majority of participants had a BMI indicative of either being overweight (27%) or obese (47.9%). This is similar to the 2016 Global Obesity Observatory’s estimate that 50% of South Africans were overweight or obese.^38^ According to WHO, globally 43% of adults are overweight and 16% are obese.^4,39^

Stigma might have played a role in 79.0% of participants not having their HIV status indicated. Of those diagnosed with HIV, the majority were on treatment. A study conducted in a sub-Saharan African setting found that the overall prevalence of MetS in people living with HIV was 21.5% compared to 12.0% in uninfected persons because of unknown mechanisms.^40^ Hypertension has also been found to be the most common MetS component in both people living with HIV and in uninfected populations.^40^

The waist circumference for the diagnosis of MetS is ≥ 80 cm for women and ≥ 94 cm for men, following the IDF Guidelines.^3^ In this study, 82.4% of participants met the waist circumference criteria for MetS. Other studies have explored different cut-offs for different ethnic groups. A study conducted in 2019 determined that the cut-off is ≥ 88 cm for women and ≥ 94 cm – 102 cm for men in Europid populations.^21^ For Mediterranean, Middle Eastern and sub-Saharan African populations, the general cut-off is accepted as ≥ 80 cm for women and ≥ 94 cm for men.^21^ A study conducted in South Africa, which focused specifically on determining the optimal waist circumference cut-off for low-income black South Africans, resulted in ≥ 89.45 cm for women and ≥ 95.25 cm for men.^22^ Considering the range of waist circumference cut-offs for different populations that have been proposed, it is worthwhile to consider an individual’s ethnic background when taking their waist circumference.^23^ A study published in 2023 aimed to redefine MetS in Africa and stressed the importance of developing African-specific criteria, which will enhance the proper diagnosis and management thereof.^37^

### Limitations

Available data were limited as some patient files were incomplete and records had to be accessed on NHLS-Labtrak (National Health Laboratory Service). It was anticipated that waist circumference measurements would not be available; therefore, the researchers measured this prospectively, following standard instructions. It was found that triglyceride, HDL cholesterol and FBG levels were not taken regularly or with each visit to the OPD; therefore, the most recent available measurements were noted for this study. It was also not possible to determine the accuracy of the FBG levels as it was unclear whether it was the fasting levels or whether the participant had eaten beforehand. As this could not be controlled, patients who had been diagnosed with type 2 diabetes were included as satisfying the FBG level inclusion criteria. Fasting blood glucose levels were mainly taken for patients already diagnosed with diabetes. The researchers could not obtain the most recent results necessary for making an accurate diagnosis of MetS. The question arises as to why triglyceride, HDL cholesterol and FBG levels are not updated regularly and controlled for patients presenting at the OPD. Possible reasons may include overburdening of hospitals and clinics, staff shortages and a lack of time per patient. The focus on one research site and the cross-sectional design afforded a snapshot of the research problem in time within a specific context. Therefore, the findings can not be generalised. Apart from the issues regarding data availability highlighted already, a number of patients refused to participate and were consequently excluded.

### Recommendations

Healthcare providers need to be educated regarding the importance of and intervals for determining triglyceride, HDL cholesterol and FBG levels. Lifestyle modifications such as healthy and affordable eating and exercise should be advocated to patients while considering their occupations and age. Human immunodeficiency virus testing of patients for whom their HIV status is unknown should be encouraged at contact with a healthcare provider. Further research needs to explore the relationship between HIV and MetS in the South African setting. Diabetes awareness campaigns should be made because a high number of patients had diabetes, but some that met the criteria were not formally diagnosed yet.

## Conclusion

Nearly a third of adult outpatient participants had insufficient information to determine their MetS status, with incomplete patient notes or special investigations not being done. Over 70% of the participants had missing information for triglyceride and HDL cholesterol levels in their patient files. Most participants did not have their HIV status stated in their patient files.

There was a high prevalence of MetS in the study, with 67.3% of the participants diagnosed, indicating a large number of patients with chronic health conditions attending this facility. Females and patients aged 60–79 years were most likely to have MetS.

## References

[1] Grundy SM, Cleeman JI, Daniels SR, et al. Diagnosis and management of the metabolic syndrome: An American Heart Association/National Heart, Lung, and Blood Institute Scientific Statement [published correction appears in Circulation. 2005;112(17):e297] [published correction appears in Circulation. 2005;112(17):e298]. Circulation. 2005;112(17):2735–2752. 10.1161/CIRCULATIONAHA.105.169404 16157765

[2] Balkau B, Charles MA. Comment on the provisional report from the WHO consultation. European Group for the Study of Insulin Resistance (EGIR). Diabet Med. 1999;16(5):442–443. 10.1046/j.1464-5491.1999.00059.x 10342346

[3] International Diabetes Foundation. The IDF consensus worldwide definition of the metabolic syndrome [document on the Internet]. Belgium: IDF; 2006 [cited 2022 Feb 25]. Available from: https://idf.org/media/uploads/2023/05/attachments-30.pdf

[4] Van Zyl S, Van der Merwe LJ, Walsh CM, Groenewald AJ, Van Rooyen FC. Risk-factor profiles for chronic diseases of lifestyle and metabolic syndrome in an urban and rural setting in South Africa. Afr J Prim Health Care Fam Med. 2012;4(1):1–10. 10.4102/phcfm.v4i1.346

[5] Bozeman SR, Hoaglin DC, Burton TM, Pashos CL, Ben-Joseph RH, Hollenbeak CS. Predicting waist circumference from body mass index. BMC Med Res Methodol. 2012;12:115. 10.1186/1471-2288-12-115 22862851 PMC3441760

[6] Harvard Health Publishing. Understanding triglycerides [homepage on the Internet]. Boston, MA: Harvard Health Publishing, Harvard Medical School; 2023 [cited 2024 Apr 11]. Available from: https://www.health.harvard.edu/heart-health/understanding-triglycerides

[7] Bhatt A. Cholesterol: Understanding HDL vs. LDL [homepage on the Internet]. Boston, MA: Harvard Health Publishing, Harvard Medical School; 2020 [cited 2024 Apr 11]. Available from: https://www.health.harvard.edu/blog/understanding-cholesterol-hdl-vs-ldl-2018041213608

[8] World Health Organization. Control your pressure, control your life [homepage on the Internet]. Geneva: WHO; 2013 [cited 2021 Jan 19]. Available from: https://www.emro.who.int/media/world-health-day/control-factsheet-2013.html

[9] Harvard Health Publishing. Rising blood sugar: How to turn it around [homepage on the Internet]. Boston, MA: Harvard Health Publishing, Harvard Medical School; 2015 [cited 2024 Apr 11]. Available from: https://www.health.harvard.edu/diabetes/rising-blood-sugar-how-to-turn-it-around

[10] Saklayen MG. The global epidemic of the metabolic syndrome. Curr Hypertens Rep. 2018;20(2):12. 10.1007/s11906-018-0812-z 29480368 PMC5866840

[11] Department of Statistics, South Africa. Mortality and causes of death in South Africa: Findings from death notification [document on the Internet]. Pretoria: StatsSA; 2017 [cited 2023 Nov 24]. Available from: https://www.statssa.gov.za/?p=14435

[12] Science Daily. Metabolic syndrome: Toxicology’s next patient. Science News. Reston, VA; Society of Toxicology; 2017 [cited 2022 Feb 16]. Available from: https://www.sciencedaily.com/releases/2017/03/170306122436.htm

[13] Tariq H, Nayudu S, Akella S, Glandt M, Chilimuri S. Non-alcoholic fatty pancreatic disease: A review of literature. Gastroenterol Res. 2016;9(6):87–91. 10.14740/gr731w PMC5191895 28058076

[14] Phillips AN, Carr A, Neuhaus J, et al. Interruption of antiretroviral therapy and risk of cardiovascular disease in persons with HIV-1 infection: Exploratory analyses from the SMART trial. Antivir Ther. 2008;13(2):177–187. 10.1177/135965350801300215 18505169

[15] Grunfeld C, Pang M, Doerrler W, Shigenaga JK, Jensen P, Feingold KR. Lipids, lipoproteins, triglyceride clearance, and cytokines in human immunodeficiency virus infection and the acquired immunodeficiency syndrome. J Clin Endocrinol Metab. 1992;74(5):1045–1052. 10.1210/jcem.74.5.1373735 1373735

[16] Asgedom YS, Kebede TM, Gebrekidan AY, et al. Prevalence of metabolic syndrome among people living with human immunodeficiency virus in sub-Saharan Africa: A systematic review and meta-analysis. Sci Rep. 2024;14:11709. 10.1038/s41598-024-62497-y 38777850 PMC11111734

[17] Nguyen KA, Peer N, De Villiers A, et al. Metabolic syndrome in people living with human immunodeficiency virus: An assessment of the prevalence and the agreement between diagnostic criteria. Int J Endocrinol. 2017;2017:1613657. 10.1155/2017/1613657 28392801 PMC5368417

[18] Willig AL, Overton ET. Metabolic complications and glucose metabolism in HIV infection: A review of the evidence. Curr HIV/AIDS Rep. 2016;13(5):289–296. 10.1007/s11904-016-0330-z 27541600 PMC5425100

[19] Guthold R, Stevens GA, Riley LM, Bull FC. Almost 40% of South Africans dangerously inactive – WHO study [homepage on the Internet]. Lansdowne: JUTA Medical Brief, Africa’s Medical Digest; 2018 [cited 2020 Jan 19]. Available from: https://www.medicalbrief.co.za/almost-40-south-africans-dangerously-inactive-study/

[20] Jiang SZ, Lu W, Zong XF, Ruan HY, Liu Y. Obesity and hypertension. Exp Ther Med. 2016;12(4):2395–2399. 10.3892/etm.2016.3667 27703502 PMC5038894

[21] Lear SA, Gasevic D. Ethnicity and metabolic syndrome: Implications for assessment, management and prevention. Nutrients. 2019;12(1):15. 10.3390/nu12010015 31861719 PMC7019432

[22] Owolabi EO, Ter Goon D, Adeniyi OV, Ajayi AI. Optimal waist circumference cut-off points for predicting metabolic syndrome among low-income black South African adults. BMC Res Notes. 2018;11(1):22. 10.1186/s13104-018-3136-9 29329600 PMC5766973

[23] Tahapary DL, Harbuwono DS, Yunir E, Soewondo P. Diagnosing metabolic syndrome in a multi-ethnic country: Is an ethnic-specific cut-off point of waist circumference needed? Nutr Diabetes. 2020;10(1):19. 10.1038/s41387-020-0123-8 32513949 PMC7280283

[24] Moore JX, Chaudhary N, Akinyemiju T. Metabolic syndrome prevalence by race/ethnicity and sex in the United States, National Health and Nutrition Examination Survey, 1988–2012. Prev Chronic Dis. 2017;14:E24. 10.5888/pcd14.160287 28301314 PMC5364735

[25] Hostinar CE, Ross KM, Chen E, Miller GE. Early-life socioeconomic disadvantage and metabolic health disparities. Psychosom Med. 2017;79(5):514–523. 10.1097/PSY.0000000000000455 28178032 PMC5453844

[26] Owolabi EO, Ter Goon D, Adeniyi OV, Adedokun AO, Seekoe E. Prevalence and correlates of metabolic syndrome among adults attending healthcare facilities in Eastern Cape, South Africa. Open Public Health J. 2017;10(1):148–159. 10.2174/1874944501710010148

[27] Gradidge PJ. Factors associated with obesity and metabolic syndrome in ageing black South African women. Glob Health Action. 2017;10(1):1359922. 10.1080/16549716.2017.1359922 29016249 PMC5645693

[28] Seloka MA, Matshipi M, Mphekgwana PM, Monyeki KD. Obesity indices to use for identifying metabolic syndrome among rural adults in South Africa. Int J Environ Res Public Health. 2020;17(22):8321. 10.3390/ijerph17228321 33187051 PMC7696649

[29] Kamerman P. Underdiagnosis of hypertension and diabetes mellitus in South Africa. S Afr Med J. 2022;112(1):53–60. 10.7196/SAMJ.2022.v112i1.15968 35140005

[30] Alberti KG, Zimmet PZ. Definition, diagnosis and classification of diabetes mellitus and its complications. Part 1: Diagnosis and classification of diabetes mellitus provisional report of a WHO consultation. Diabet Med. 1998;15:539–553. 10.1002/(SICI)1096-9136(199807)15:7<539::AID-DIA668>3.0.CO;2-S 9686693

[31] Akinyemiju T, Chaudhary N, Moore JX. Metabolic syndrome prevalence by race/ ethnicity and sex in the United States, National Health and Nutrition Examination Survey, 1988–2012. Prev Chronic Dis. 2017;14:160287. 10.5888/pcd14.160287 PMC5364735 28301314

[32] Kruger MJ, Nell TA. The prevalence of the metabolic syndrome in a farm worker community in the Boland district, South Africa. BMC Public Health. 2017;17(1):61. 10.1186/s12889-016-3973-1 28077105 PMC5225561

[33] O’Neill A. South Africa: Total population from 2012 to 2022, by gender [homepage on the Internet]. New York, NY: Statista; 2024 [cited 2024 Apr 11]. Available from: https://www.statista.com/statistics/967928/total-population-of-south-africa-by-gender/

[34] Statistics South Africa. People [homepage on the Internet]. Statistics South Africa. Statssa.gov.za; 2019 [cited 2022 Feb 25]. Available from: http://www.statssa.gov.za/?page_id=737&id=1

[35] Meloni A, Cadeddu C, Cugusi L, et al. Gender differences and cardiometabolic risk: The importance of the risk factors. Int J Mol Sci. 2023;24(2):1588. 10.3390/ijms24021588 36675097 PMC9864423

[36] Hildrum B, Mykletun A, Hole T, et al. Age-specific prevalence of the metabolic syndrome defined by the International Diabetes Federation and the National Cholesterol Education Program: The Norwegian HUNT 2 study. BMC Public Health. 2007;7:220. 10.1186/1471-2458-7-220 17727697 PMC2048947

[37] Charles-Davies MA, Ajayi OO. Redefining the metabolic syndrome in Africa: A systematic review between 2005 and 2022. Dubai Diabetes Endocrinol J. 2023;29(2):89–98. 10.1159/000531552

[38] World Obesity. Global Obesity Observatory. South Africa, adults, 2016 [homepage on the Internet]. 2016 [cited 2022 Feb 23]. Available from: https://data.worldobesity.org/country/south-africa-197/#data_prevalence

[39] World Health Organization. Obesity and overweight [homepage on the Internet]. WHO. [cited 2024 Jul 08; updated 2024 Jul 03]. Available from: https://www.who.int/news-room/fact-sheets/detail/obesity-and-overweight#:~:text=The%20diagnosis%20of%20overweight%20andd

[40] Todowede OO, Mianda SZ, Sartorius B. Prevalence of metabolic syndrome among HIV-positive and HIV-negative populations in sub-Saharan Africa-a systematic review and meta-analysis. Syst Rev. 2019;8(1):4. 10.1186/s13643-018-0927-y 30606249 PMC6317235

